# Data triangulation to estimate age-specific coverage of voluntary medical male circumcision for HIV prevention in four Kenyan counties

**DOI:** 10.1371/journal.pone.0209385

**Published:** 2018-12-18

**Authors:** Katharine Kripke, Marjorie Opuni, Elijah Odoyo-June, Mathews Onyango, Peter Young, Kennedy Serrem, Vincent Ojiambo, Melissa Schnure, Peter Stegman, Emmanuel Njeuhmeli

**Affiliations:** 1 Avenir Health, Project SOAR, Glastonbury, CT, United States of America; 2 Independent consultant, Geneva, Switzerland; 3 Division of Global AIDS and TB, US CDC, Nairobi, Kenya; 4 FHI 360, Nairobi, Kenya; 5 NASCOP, Nairobi, Kenya; 6 USAID, Nairobi, Kenya; 7 Palladium Group, Project SOAR, Washington, D.C., United States of America; 8 USAID, Mbabane, Swaziland; Weill Cornell Medical College, UNITED STATES

## Abstract

**Background:**

Kenya is 1 of 14 priority countries in Africa scaling up voluntary medical male circumcision (VMMC) for HIV prevention following the recommendations of the World Health Organization and the Joint United Nations Programme on HIV/AIDS. To inform VMMC target setting, we modeled the impact of circumcising specific client age groups across several Kenyan geographic areas.

**Methods:**

The Decision Makers’ Program Planning Tool, Version 2 (DMPPT 2) was applied in Kisumu, Siaya, Homa Bay, and Migori counties. Initial modeling done in mid-2016 showed coverage estimates above 100% in age groups and geographic areas where demand for VMMC continued to be high. On the basis of information obtained from country policy makers and VMMC program implementers, we adjusted circumcision coverage for duplicate reporting, county-level population estimates, migration across county boundaries for VMMC services, and replacement of traditional circumcision with circumcisions in the VMMC program. To address residual inflated coverage following these adjustments we applied county-specific correction factors computed by triangulating model results with coverage estimates from population surveys.

**Results:**

A program record review identified duplicate reporting in Homa Bay, Kisumu, and Siaya. Using county population estimates from the Kenya National Bureau of Statistics, we found that adjusting for migration and correcting for replacement of traditional circumcision with VMMC led to lower estimates of 2016 male circumcision coverage especially for Kisumu, Migori, and Siaya. Even after addressing these issues, overestimation of 2016 male circumcision coverage persisted, especially in Homa Bay. We estimated male circumcision coverage in 2016 by applying correction factors. Modeled estimates for 2016 circumcision coverage for the 10- to 14-year age group ranged from 50% in Homa Bay to approximately 90% in Kisumu. Results for the 15- to 19-year age group suggest almost complete coverage in Kisumu, Migori, and Siaya. Coverage for the 20- to 24-year age group ranged from about 80% in Siaya to about 90% in Homa Bay, coverage for those aged 25–29 years ranged from about 60% in Siaya to 80% in Migori, and coverage in those aged 30–34 years ranged from about 50% in Siaya to about 70% in Migori.

**Conclusions:**

Our analysis points to solutions for some of the data issues encountered in Kenya. Kenya is the first country in which these data issues have been encountered because baseline circumcision rates were high. We anticipate that some of the modeling methods we developed for Kenya will be applicable in other countries.

## Introduction

Voluntary medical male circumcision (VMMC) is an effective intervention to reduce female-to-male HIV transmission [1–3]. Kenya is one of 14 priority countries in Eastern and Southern Africa that are scaling up VMMC for HIV prevention following the recommendations of the World Health Organization (WHO) and the Joint United Nations Programme on HIV/AIDS (UNAIDS) [4]. The VMMC program in Kenya started at the end of 2008 with the publication of the National Guidance on Male Circumcision [5] and the first Kenya National Strategy for Voluntary Medical Male Circumcision issued in 2009 [6]. Implementation of the VMMC program began in the Nyanza region (the region where male circumcision was lowest and HIV prevalence was highest) and was then expanded to other regions, including Nairobi, parts of Rift Valley and Western regions [7]. Given the high prevalence of traditional circumcision in many parts of Kenya, the initial target of the VMMC program was to increase the proportion of circumcised men aged 15–49 years by 2013 from 85% to 94% nationally and from 48% to 80% in Nyanza [7]. Data from the 2014 Kenya Demographic and Health Survey (KDHS) showed that the proportion of men aged 15–49 years in Kenya who were circumcised increased to 93% and the proportion in Nyanza increased to 72% [8].The Government of Kenya has long been committed to optimizing the impact of HIV resources by scaling up evidence-based interventions and focusing on appropriate populations and geographic areas to minimize new HIV infections and AIDS-related deaths [9–11]. With the devolution of government functions, Nyanza province was split into six counties (Kisii, Nyamira, Kisumu, Siaya, Homa Bay, and Migori). The second National Strategy for Voluntary Medical Male Circumcision covering 2014/15 to 2018/19 has prioritized VMMC scale up among men aged 15–49 years in Kisumu, Siaya, Homa Bay, and Migori, based on high HIV prevalence and high proportion of uncircumcised men [7]. The strategy also prioritizes VMMC service provision in counties where pockets of non-circumcised subgroups exist even though the overall circumcision rate is high (Nairobi, Busia, Kericho, Nandi, Turkana, and West Pokot).

Several recent studies indicate that increasing the number of circumcised men aged 15–29 years will produce the most immediate impact on HIV incidence compared to circumcising other age groups [12–21]. In addition, evidence suggests that boys aged 10–14 years should also be included in the targets because they are key for maintaining saturation once achieved and have high intrinsic demand for VMMC; coming in for services regardless of the age focus of demand creation interventions [17, 22].

To inform policy discussions on age targeting in the VMMC program in Kenya, modeling with the Decision Makers’ Program Planning Tool, Version 2 (DMPPT 2) was undertaken in mid-2016. The DMPPT 2 was applied to assess the impact of circumcising specific age groups across geographic areas. This initial modeling showed male circumcision coverage greater than 100% in certain age groups and geographic areas where demand for VMMC continued to be high. To inform VMMC target setting in Kenya, we used data cleaning and triangulation to resolve the inconsistencies in the initial DMPPT 2 male circumcision coverage estimates. This paper describes the data correction and triangulation undertaken to estimate age-specific coverage of male circumcision for HIV prevention in four Kenyan counties (Kisumu, Siaya, Homa Bay, and Migori). Our findings highlight the importance of disaggregating data by age groups to understand male circumcision coverage trends to inform program planning. They also provide useful information for other countries facing similar VMMC data quality issues.

## Methods

### Ethics

This study was determined by the Institutional Review Board of the Population Council to be exempt from review on January 6, 2016. The data used in this study included publicly available data and routine program data. All program data were fully anonymized prior to provision to the researchers. No medical records were reviewed as part of this research.

### DMMPT 2 model

The DMPPT 2 model has been described in detail elsewhere [17, 19]. Briefly, DMPPT 2 is a simple, compartmental model implemented in Microsoft Excel 2010 that was designed to analyze the effects of age at circumcision on program impact and cost-effectiveness. The model tracks the number of circumcised males in newborns and in each 5-year age group over time, taking into account age progression and mortality. The model also calculates discounted VMMC program costs and HIV infections averted in the population in each year in a user-specified VMMC scale-up strategy. These are compared to a baseline scenario in which male circumcision prevalence is held constant at the level found before the initiation of VMMC services for HIV prevention.

### Data used for initial DMPPT 2 modeling

Separate DMPPT 2 model files were created for each of the four counties. For the initial DMPPT 2 modeling we exported population, mortality, and HIV incidence and prevalence projections from four county-level Spectrum/Goals files [23] that had been assembled in consultation with country policy makers and implementers for a separate exercise comparing three different models to estimate the impact of the VMMC program in Kenya. The Goals model within the Spectrum suite of models is a compartmental deterministic model incorporating demography, HIV epidemiology, sexual behavior, HIV disease progression, and the impact of HIV treatment and prevention interventions on mortality and new HIV infections.

The DMPPT 2 model requires as an input the baseline male circumcision prevalence before scale-up of VMMC. However, whereas the Kenya VMMC program began at the end of 2008, household surveys collecting information on self-reported male circumcision status such as the Kenya AIDS Indicator Survey (KAIS) and the KDHS were not powered to the county level before 2012. The baseline male circumcision prevalence for each county was therefore estimated by

$$\sum_{i=1}^{n}c_{i}\times e_{i,j}$$

- Where *c* is the male circumcision prevalence in ethnic group *i* (ages 15–49) as reported in the 2008 KDHS [24] before significant implementation of VMMC, and
- *e* is the prevalence of ethnic group *i* in county *j* as reported in the 2014 KDHS [8].
- *n* is the number of ethnic groups assessed in the 2008 KDHS.

The distribution of ethnic groups in each county was assumed not to change substantially between 2008 and 2014.

The research team compiled age-disaggregated VMMC numbers from the beginning of the program through September 2016 as follows. In August 2016, the Ministry of Health provided the numbers of VMMCs conducted in each county from the beginning of the program in 2008 through the end of December 2015 as per their program records. In September 2016, the Ministry of Health also provided a de-identified subset of VMMC client records with information on client age which was captured at the time of circumcision, totalling 325,000. A data quality assessment comparing digital records with paper files at VMMC sites was conducted for these 325,000 records from Homa Bay, Kisumu, Migori, and Siaya covering years 2012–2015. The age distribution by county for these 325,000 records was applied to the total number of VMMCs reported per county for the previous years because the Ministry of Health deemed reported client ages unreliable prior to 2013. Age-disaggregated VMMC numbers for 1 January–30 September 2016 for each county were provided by the U.S. President’s Emergency Plan for AIDS Relief (PEPFAR) team in Kenya in January 2017. The numbers were provided through 30 September 2016, so that the modeling team could assist with PEPFAR VMMC program planning based on the U.S. government fiscal year.

### Issues with VMMC data in Kenya

As discussed below, the initial DMPPT 2 modeling for Homa Bay, Kisumu, Migori, and Siaya resulted in greater than 100% estimated male circumcision coverage in certain age groups and geographic areas at the end of 2016 even though demand for VMMC persisted in all age groups in the four counties. The following data issues were identified as possible drivers in consultation with the country team comprised of country policy makers and VMMC program implementers.

First, duplicate reporting of male circumcisions was identified as one possible source of inflated coverage numbers, with PEPFAR implementing partners perhaps inadvertently reporting on a circumcision more than once or both implementing partners and their subcontractors reporting on the same circumcision.

Second, the use of United Nations (UN) Population Division national population data in the initial DMPPT 2 modeling was questioned with concerns raised as to the accuracy of county-level population estimates over time by age group that impacted the age-specific male circumcision coverage estimates coming out of the model. Country policy makers and VMMC program implementers advised that county population estimates from the Kenya National Bureau of Statistics (KNBS) should be used as inputs into county-level models on VMMC instead of the UN Population Division estimates disaggregated to the county level. Whereas the UN Population Division-based population estimates were derived by disaggregating the national population and applying national fertility and mortality estimates at the county level, the KNBS county population estimates were calibrated more precisely to the demographics of each county.

Migration across county boundaries for VMMC services was identified as a third factor that might contribute to the inflated coverage estimates. Data from Kenya suggest the existence of substantial cross-county migration [25, 26]. This mobility was hypothesized to result in individuals undergoing circumcision in a county other than their county of residence. If enough clients in a certain age group residing elsewhere undergo circumcision in a given county, this could contribute to coverage estimates above 100% because some of the individuals included in the numerator—the male population circumcised in a given age group—are not included in the denominator—the male population within the given age group that is not circumcised.

Fourth, the replacement of traditional circumcision with circumcisions in the VMMC program was believed to play a part in the inflated male circumcision coverage projections. The DMPPT model assumes that the background rate of traditional circumcision remains constant over time. Data from Kenya suggest that some proportion of individuals who would have undergone traditional circumcision before the scale up of VMMC are now undergoing VMMC instead of traditional circumcision; not adjusting the model inputs accordingly could lead to inflated coverage estimates.

### Correcting for duplicate reporting of VMMC

The Kenya country team reviewed VMMC program numbers reported by implementing partners for Homa Bay, Kisumu, and Siaya for 2008–2010. Following this review, the team realized that a prime partner and their subcontractor had both reported the same circumcisions for these years, and that over 48,000 circumcisions had been reported twice (see S1 Table). The country team confirmed the double counting with the implementers and identified which circumcisions were double-reported and eliminated them.

### Correcting for population, migration, and replacement

County population estimates were realigned with KNBS estimates versus UN Population Division estimates. The age distribution of the KNBS county population estimates was more heavily weighted toward the younger age groups than that of the county population estimates derived from the national UN Population Division estimates. For the years for which it was available, the age- and county-specific population estimates from KNBS were input directly into the model. For the years not included in the KNBS estimates, the county population was imputed using linear interpolation between the KNBS-estimated values.

To address migration and replacement as potential contributing factors to the inflated male circumcision coverage estimates, VMMC program staff randomly selected program reports from 100 clients per partner per county for each year during which the partner was operational through 2016. One of the implementing partners included reports from all of their clients rather than a sample. A breakdown of the percent of circumcisions conducted by each partner in each county in each year was also provided so that the client residence numbers could be weighted by the proportion of circumcisions conducted by each partner in each county. For each reviewed client report, information on the client’s county of residence was noted to determine the percent of VMMC clients in each county that were a resident in that same county. Each client’s name was also reviewed to assess whether the client was likely to be from a traditionally circumcising community or not using name as proxy for ethnic group. Each VMMC program implementer provided the researchers with percentages of their clients in each county that were judged to be from traditionally circumcising communities, and percentages of clients in each county that were resident in that county. The reported total VMMC client numbers for each county for each year of implementation were then multiplied by the percentage of clients who were both resident in that county and from traditionally non-circumcising communities (see S2 and S3 Tables).

### Addressing residual inflated coverage

Following these adjustments to address known duplicate reporting of VMMCs by partners, inaccurate population estimates, and issues with migration and replacement of traditional circumcision with VMMC, we generated correction factors to adjust for male circumcision coverage greater than 100% still projected in some age groups in each county, particularly the 15- to 19-year age group. Country policy makers and VMMC program implementers suspected that this was due to possibly intentional inflation of program reporting by some implementing partners, but we were unable to determine the source of this inflation.

The correction factor was a percentage less than 100% by which all program numbers in each year of implementation were multiplied. For Homa Bay and Kisumu, the correction factor was set manually by triangulating coverage estimates from the 2012 KAIS and the 2014 KDHS such that the model produced circumcision coverage among 15- to 49-year-olds that aligned as closely as possible with the coverage in this same age group measured in the two surveys. For Migori and Siaya, the coverage among 15- to 49-year-olds in the model was already lower than the measured coverage as reported in the two surveys, but the modeled coverage among 15- to 19-year-olds at the end of September 2016 was still above 100%. A small correction factor was applied to bring the coverage in this age group just below 100% as of September 2016.

### Impact of data adjustments on estimates of HIV infections averted

To illustrate the ways in which the various data adjustments affected DMPPT 2 modeling outputs, we also estimated the HIV infections averted from 2008 to 2020 for Homa Bay, Kisumu, Migori, and Siaya using four different male circumcision prevalence trend estimates. In Scenario A, we used unadjusted male circumcision prevalence. In Scenario B, we used adjusted male circumcision prevalence correcting for duplicate reporting. In Scenario C, we used adjusted male circumcision prevalence correcting for duplicate reporting, population, migration, and replacement. In Scenario D, we used adjusted modeled male circumcision prevalence correcting for duplicate reporting, population, migration, and replacement as well as applying the correction factor described above.

## Results

**Fig 1:**
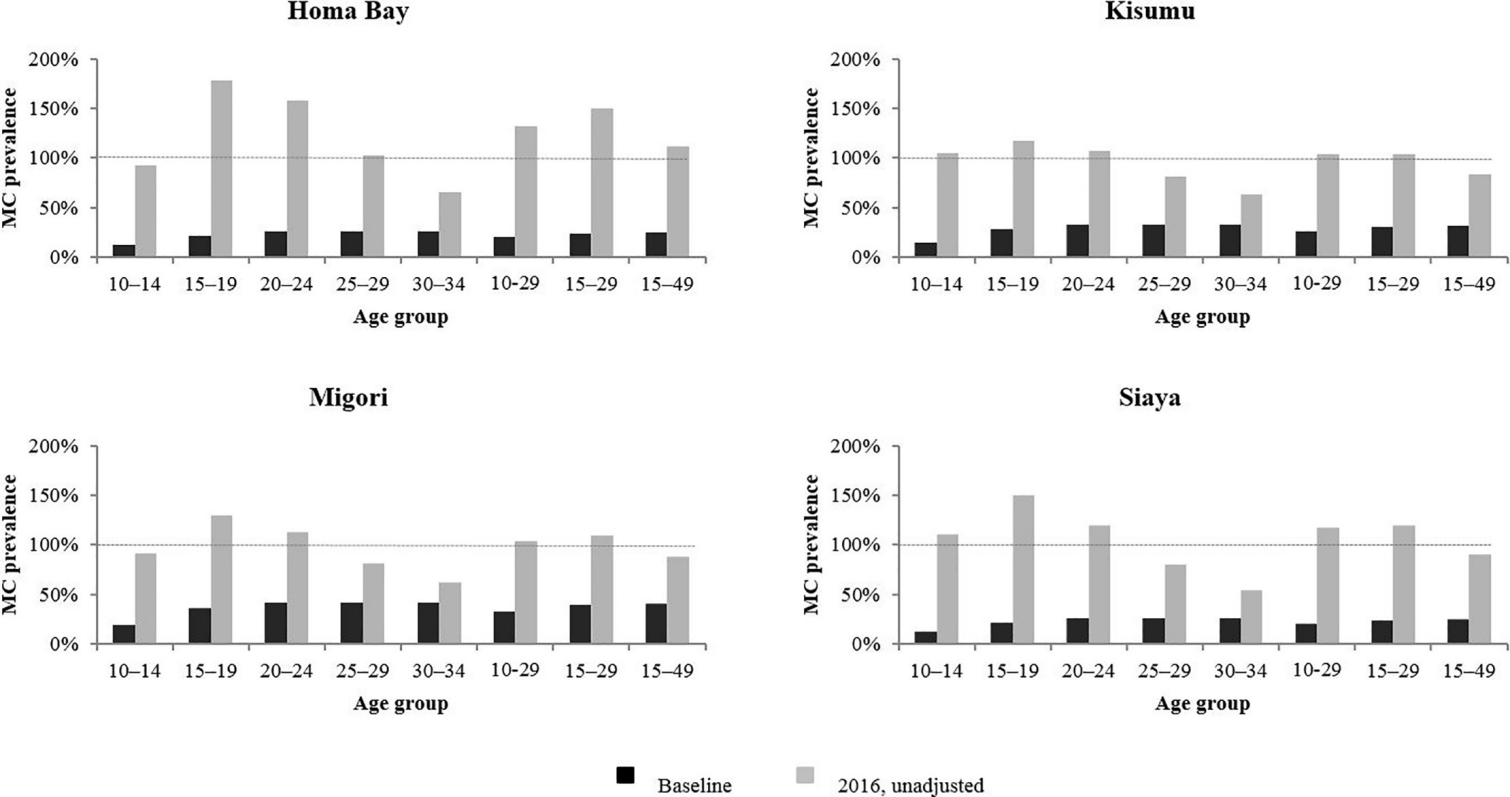
Male circumcision (MC) prevalence before start of voluntary medical male circumcision (VMMC) program and unadjusted modeled MC prevalence by end 2016 for Homa Bay, Kisumu, Migori, and Siaya. Black bars represent age specific MC prevalence before the start of the VMMC program. Light grey bars represent modeled age specific MC prevalence by end 2016. Dashed horizontal line denotes MC prevalence of 100%.

Fig 1 shows the results of the initial application of the DMPPT 2 to assess male circumcision coverage among specific client age groups in Homa Bay, Kisumu, Migori, and Siaya counties. Whereas projections of male circumcision prevalence for all 15- to 49-year-olds using data available in March 2016 resulted in coverage estimates for end of 2016 that were above 100% only in Homa Bay, this initial modeling showed male circumcision coverage estimates above 100% in certain age groups in all four counties. In Homa Bay, modeled estimates for circumcision prevalence for end 2016 were almost 180% for 15- to 19-year-olds, almost 160% for 20- to 24-year-olds, and just over 100% for men aged 25–29 years. In Kisumu, Migori, and Siaya, modeled estimates for end 2016 were well over 100% for 15- to 19-year-olds and 20- to 24-year-olds; and in Kisumu and Siaya, end 2016 estimates were also above 100% for boys aged 10–14 years.

**Fig 2:**
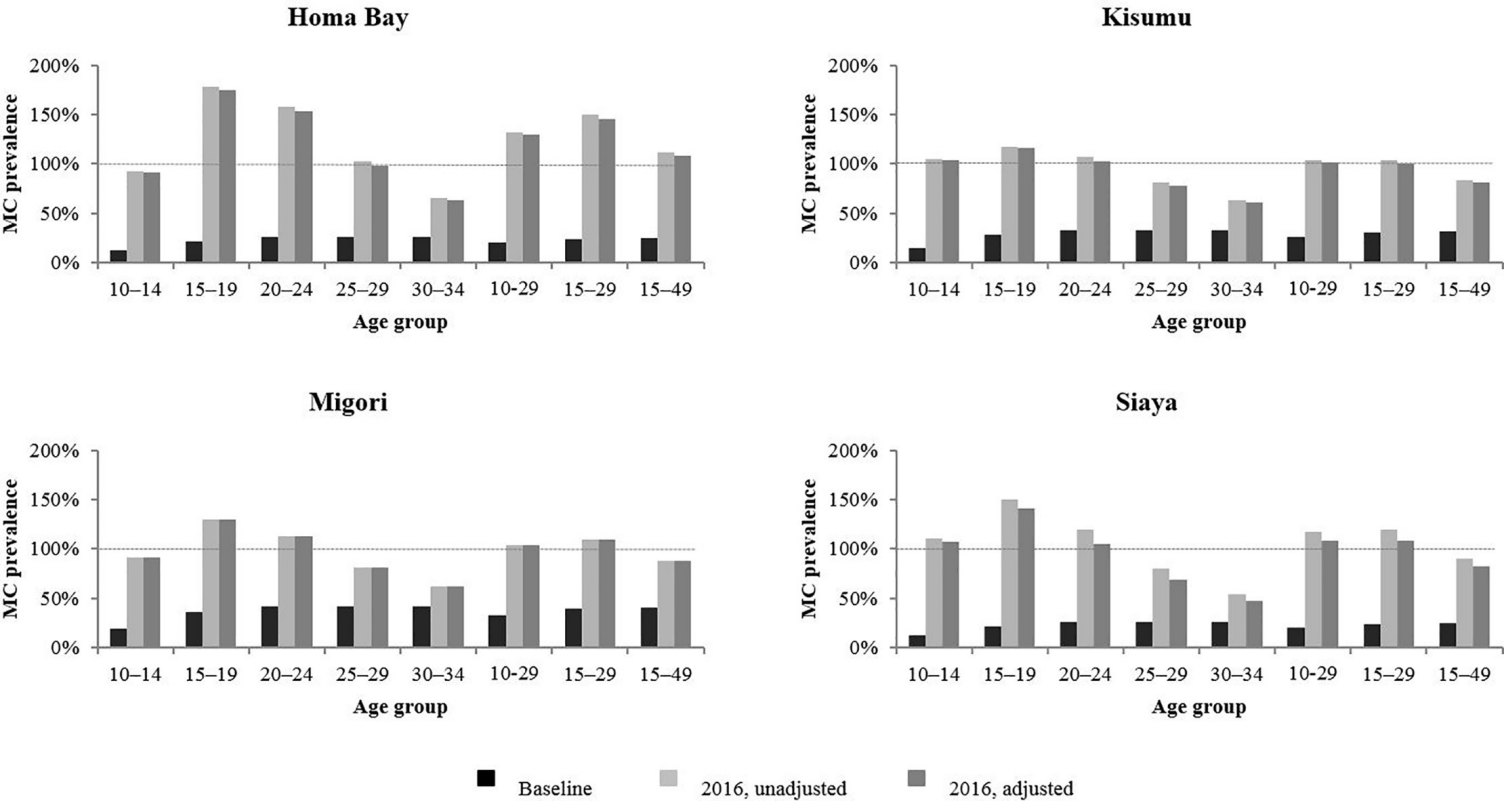
Male circumcision (MC) prevalence before start of voluntary medical male circumcision (VMMC) program, unadjusted modeled MC prevalence by end 2016, and adjusted modeled MC prevalence by end 2016 correcting for duplicate reporting for Homa Bay, Kisumu, Migori, and Siaya. Black bars represent age specific MC prevalence before the start of the VMMC program. Light grey bars represent unadjusted modeled age specific MC prevalence by end 2016. Dark grey bars represent adjusted modeled age specific MC prevalence by end 2016 correcting for duplicate reporting. Dashed horizontal line denotes MC prevalence of 100%.

Fig 2 shows baseline circumcision prevalence rates in 2008, unadjusted male circumcision prevalence rates by end 2016, and adjusted male circumcision prevalence rates after correcting for duplicate reporting of VMMCs by implementing partners. This figure shows that duplicate reporting was a small contributing factor to the inflated 2016 circumcision coverage estimates in Homa Bay, Kisumu, and Siaya.

**Fig 3:**
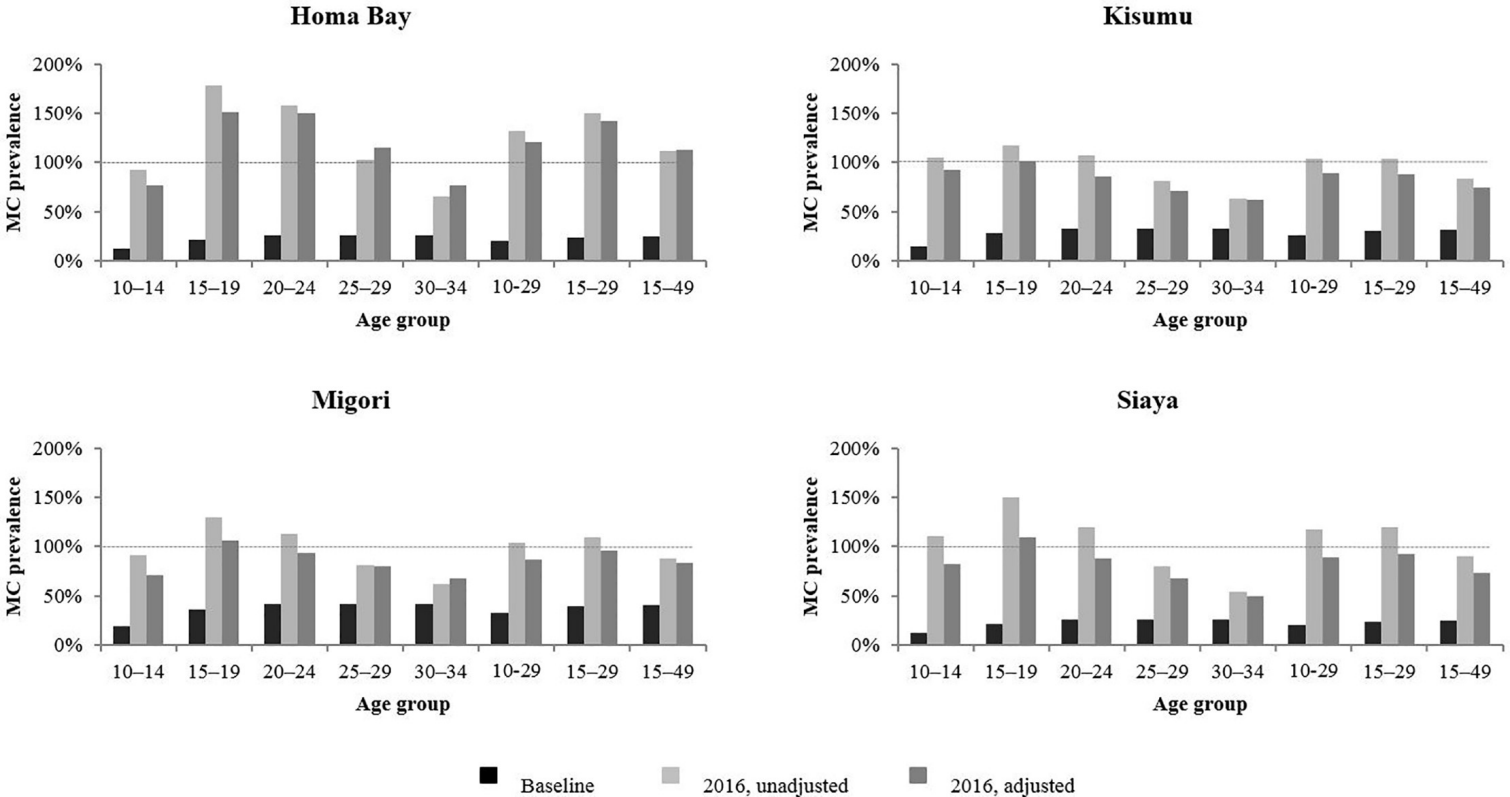
Male circumcision (MC) prevalence before start of voluntary medical male circumcision (VMMC) program, unadjusted modeled MC prevalence by end 2016, and adjusted modeled MC prevalence by end 2016 correcting for duplicate reporting, population, migration, replacement for Homa Bay, Kisumu, Migori, Siaya. Black bars represent age specific MC prevalence before the start of the VMMC program. Light grey bars represent unadjusted modeled age specific MC prevalence by end 2016. Dark grey bars represent adjusted modeled age specific MC prevalence by end 2016 correcting for duplicate reporting, population, migration, and replacement. Dashed horizontal line denotes MC prevalence of 100%.

Fig 3 shows the effect of correcting for duplicate reporting, population, migration, and replacement. Correcting for duplicate reporting of VMMCs, aligning county population estimates with KNBS data, and adjusting program data for migration as well as the replacement of traditional circumcision by VMMC led to much lower estimates of 2016 male circumcision coverage, especially for Kisumu, Migori, and Siaya.

However, even after addressing all of these possible contributing factors, the DMPPT 2 estimates for 2016 circumcision coverage were above 100% for men aged 15–19 years old, 20–24 years old, and 25–29 years old in Homa Bay as well as for men aged 15–19 years in Kisumu, Migori, and Siaya. Table 1 shows baseline male circumcision prevalence for Homa Bay, Kisumu, Migori, and Siaya as well as male circumcision prevalence for the four counties according to the 2012 KAIS and the 2014 KDHS. Table 1 also shows the correction factors applied to program data from each county to account for the residual inflation in coverage estimates that was unaccounted for after correcting for duplicate reporting, population, migration, and replacement. Finally, it compares the DMPPT 2 unadjusted and adjusted coverage estimates for each county in 2012 and 2014 to the coverage levels obtained in the population surveys. Table 1 further highlights that after correcting for known or suspected factors, inflated estimation of coverage over time with the DMPPT 2 was a particular concern in Homa Bay; coverage estimates for Kisumu, Migori, and Siaya were more closely aligned with population survey results.

**Table 1:** Triangulation of coverage estimates and correction factors for Homa Bay, Kisumu, Migori, and Siaya.

Year		Homa Bay %	Kisumu %	Migori %	Siaya %
	**Correction factor**^**a**^	54	92	91	89
2008	Baseline MC prevalence	24.9	32.2	41.0	25.2
2012	KAIS 2012	41	45	61	45
	DMPPT 2 unadjusted coverage	54.2	49.1	52.8	42.0
	DMPPT 2 adjusted coverage	41.4	47.8	51.9	40.3
2014	KDHS 2014	56.0	58.8	72.5	55.9
	DMPPT 2 unadjusted coverage	81.1	60.2	66.0	54.4
	DMPPT 2 adjusted coverage	55.9	58.0	63.9	51.3
2016	DMPPT 2 adjusted, ages 15–19 years	94.0	95.7	99.4	99.7

Abbreviations: MC, male circumcisions; KAIS, Kenya AIDS Indicator Survey; DMPPT, Decision Makers’ Program Planning Tool; KDHS, Kenya Demographic and Health Survey

^**a**^Correction factor applies equally to all reported circumcisions, regardless of age.

**Fig 4:**
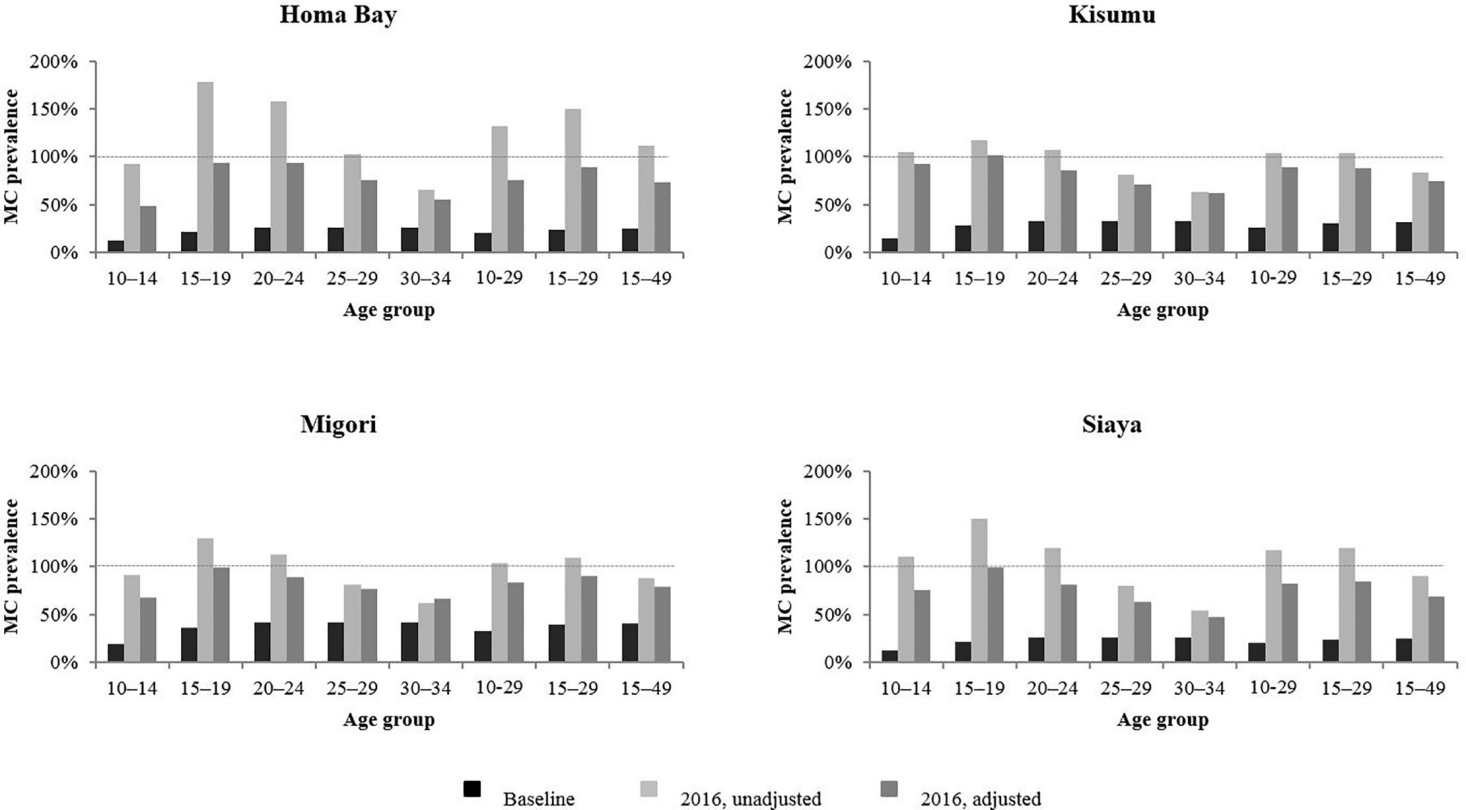
Male circumcision (MC) prevalence before start of voluntary medical male circumcision (VMMC) program, unadjusted modeled MC prevalence by end 2016, and adjusted modeled MC prevalence by end 2016 correcting for duplicate reporting, population, migration, replacement, and applying county-specific correction factors for Homa Bay, Kisumu, Migori, and Siaya. Black bars represent age specific MC prevalence before the start of the VMMC program. Light grey bars represent unadjusted modeled age specific MC prevalence by end 2016. Dark grey bars represent adjusted modeled age specific MC prevalence by end 2016 correcting for duplicate reporting, population, migration, and replacement as well as applying a country-specific correction factor. Dashed horizontal line denotes male circumcision prevalence of 100%.

The results of the final application of the DMPPT 2 incorporating the full set of adjustments are shown in Fig 4. Correcting for duplicate reporting, population size, migration, replacement, and applying the correction factors shown in Table 1, modeled estimates for 2016 circumcision coverage for 10- to 14-year-olds ranged from 50% in Homa Bay to approximately 90% in Kisumu. Results for 15- to 19-year-olds suggest almost complete coverage in Kisumu, Migori, and Siaya. Coverage for 20- to 24-year age groups ranges from about 80% in Siaya to about 90% in Homa Bay, coverage for those aged 25–29 years ranges from about 60% in Siaya to 80% in Migori, and coverage in those aged 30–34 years ranges from about 50% in Siaya to about 70% in Migori.

**Fig 5:**
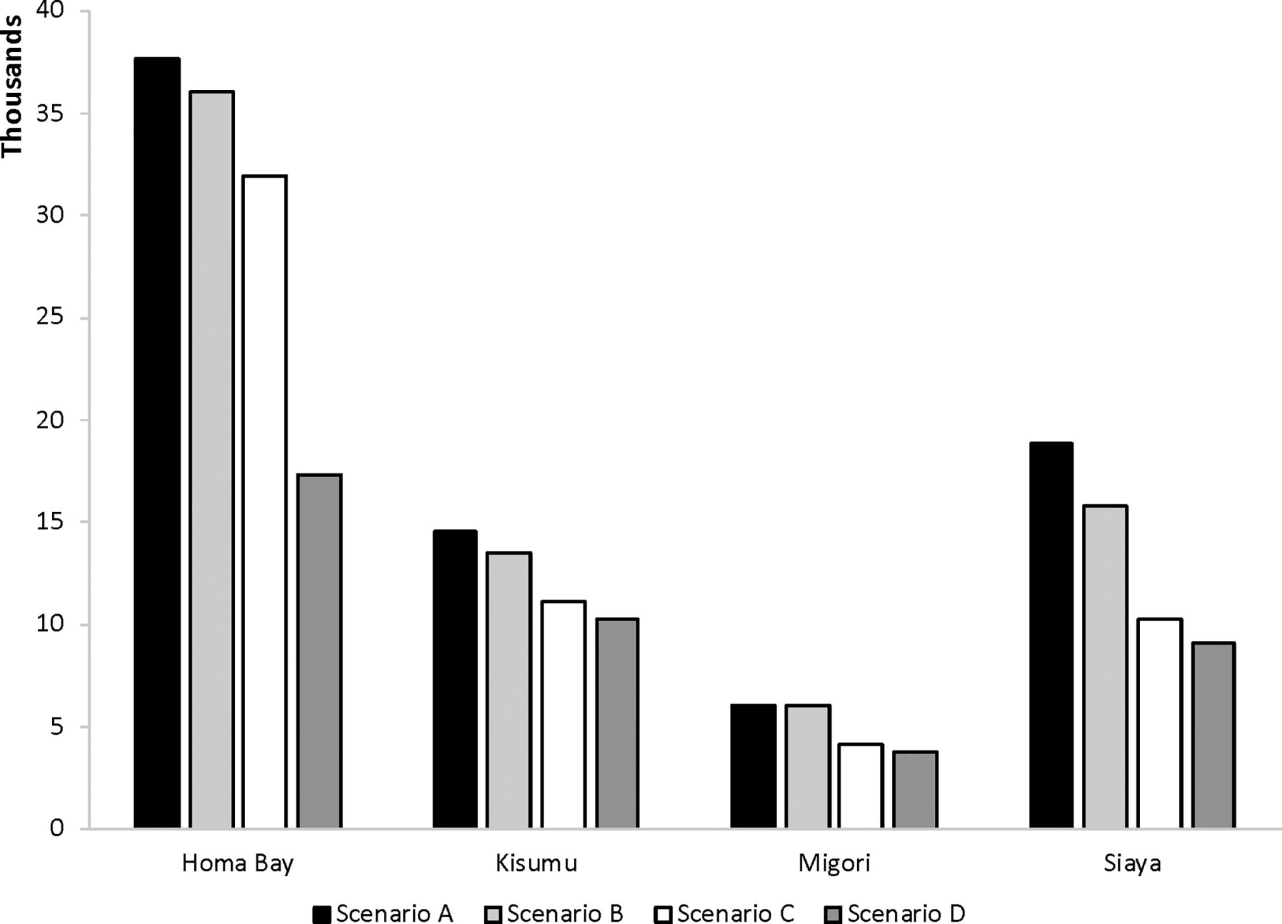
HIV infections averted in Homa Bay, Kisumu, Migori, and Siaya, 2008–2020. Estimates of HIV infections averted by county from 2008–2020 using unadjusted male circumcision (MC) prevalence estimates (Scenario A), using MC prevalence estimates corrected for duplicate reporting alone (Scenario B), using MC prevalence estimates corrected for duplicate reporting, population, migration, and replacement (Scenario C), and using MC prevalence estimates corrected for duplicate reporting, population, migration, and replacement and with an applied correction factor (Scenario D).

The impact of the data adjustments on the DMPPT 2 modeling outputs are illustrated in Fig 5 which shows the estimates of HIV infections averted for each of the four counties from 2008 to 2020 using unadjusted male circumcision prevalence as well as the male circumcision coverage estimates corrected for duplicate reporting alone; corrected for duplicate reporting, population, migration, and replacement; and corrected for duplicate reporting, population, migration, and replacement and with a county-specific correction factor (See S1 Appendix for the estimated number of VMMCs for 2008–2017 in the four scenarios). Whereas the estimated number of HIV infections averted from 2008 to 2020 ranged from around 6,000 in Migori to almost 40,000 in Homa Bay when male circumcision numbers were unadjusted (with male circumcision coverage estimates above 100%), these ranged from around 4,000 in Migori to almost 18,000 in Homa Bay when male circumcision coverage was adjusted for all of the factors identified.

## Discussion

Our modeling analysis results underscore the importance of disaggregating male circumcision coverage estimates by age groups to understand patterns of coverage and to inform program planning and implementation. Our findings show that analyzing data at a more granular level focusing on subnational areas and subpopulations within counties can provide additional insights into data quality and program implementation progress. We also describe some novel processes and approaches that can be used to address these issues in other countries.

Modeling age-specific VMMC coverage in Homa Bay, Kisumu, Migori, and Siaya counties between 2008 and 2016, we identified important limitations in the available VMMC program data. These issues varied somewhat by geographic area. We identified duplicate reporting as a contributing factor in Homa Bay, Kisumu, and Siaya. Using county population estimates from the Kenya National Bureau of Statistics, adjusting for migration and correcting for replacement of traditional circumcision with VMMC led to much lower estimates of 2016 male circumcision coverage especially for Kisumu, Migori, and Siaya. Even after addressing these issues, overestimation of 2016 male circumcision coverage estimates persisted—especially in Homa Bay. We estimated male circumcision coverage in 2016 after applying a correction factor computed by triangulating model results with coverage estimates from population surveys.

The fact that we had to apply such a correction factor and that it had to be of such magnitude in Homa Bay does raise an important question: what underlies the inflation in coverage estimates persisting even after adjusting for what the country team identified as potential contributing factors? Several factors for which we were unable to adjust include lack of precision in the age-specific baseline circumcision estimates and misreporting of client ages. Also, this enduring inflation suggests that program data may have been over reported by facilities over time, especially in Homa Bay. Although there is no conclusive evidence of deliberate over-reporting, the country team noted discrepancies between the numbers of circumcisions recorded in the site registers and the numbers reported in the facility aggregation forms and uploaded in the district health information system.

The purpose of our study was to help the country team identify how many clients in each age group would need to be circumcised in each county annually in order to reach and maintain coverage targets. A number of policy and programming recommendations arise from this analysis. Our results suggest that household population surveys and special male circumcision prevalence surveys that collect information on self-reported male circumcision status should include a question about age at circumcision and should, if possible, also include large enough samples to generate estimates of male circumcision coverage by age bands and subnational units with sufficient power. Valid client contact information—including residence—should be included in program records, as lack of these details makes spot quality checks difficult. Collecting information on whether clients come from traditionally circumcising communities, such as whether parents were circumcised traditionally, would also be helpful. Our findings also illustrate the importance of data quality assessments of clinic records is also made evident. Ongoing data quality assessment exercises including spot checks would be important—especially in Homa Bay.

Kenya is the first country in which we encountered these data issues because the baseline level of circumcision is high. We were able to explore these data issues in Kenya because the country has relatively rich and detailed data (e.g., client residence and the ability to estimate replacement of traditional circumcision using client names as a proxy for ethnicity). Applying the DMMPT 2 model in other countries, we have encountered a few other cases of saturated circumcision coverage including in certain age groups in some districts in Mozambique and South Africa. We anticipate that the methods we developed for Kenya will also be applicable in other countries, depending on data availability.

Our study has several limitations. The limitations of the DMPPT 2 model have been described elsewhere [19]. Our adjustment approach to address replacement of traditional circumcision with VMMC is conservative and assumes that all clients from circumcising communities would have been circumcised in the absence of the VMMC program, which might lead to overestimation of the extent of replacement. Our adjustment method is also limited by the fact that some names are shared across ethnic groups, and intermarriage may confound the determination of whether someone would undergo traditional circumcision in the absence of the VMMC program. We also assumed that the age distribution of clients was constant in the years prior to 2014, and this could contribute to inflated coverage estimates in the younger age groups if the initial clients in the program were actually older and the later clients were younger. We also assumed that ethnic distribution was constant over time using the 2014 KDHS ethnic distribution.

The greatest source of uncertainty in this analysis is related to the correction factor. The correction factor was applied equally to all age groups and all years, but since the source of the discrepancy is still unknown, there is no way of knowing whether the correction factor should have been applied differently to different age groups and different years. For Homa Bay, there may have been some systematic source of the discrepancy, given that the correction factor was substantial and that it was possible to match both surveys using a single correction factor. For the other counties, the need for correction was substantially less, and the excess estimated male circumcision coverage is plausibly in the range of the error of estimating the baseline circumcision coverage. For Migori and Siaya, the modeled coverage estimates were lower than the male circumcision coverage measured in the household surveys, but a correction factor was still needed to keep the coverage among 15- to 19-year-olds below 100%. The actual coverage in this age group will remain unknown until it is measured using an age-specific survey, so it is difficult to use the adjusted numbers to project program targets.

Despite these limitations, this analysis highlights the importance of age-disaggregated data to understand VMMC coverage trends to inform program planning. The major utility of the analysis is that it points to solutions for some of the data issues encountered in Kenya. Before new data becomes available for Kenya, these estimates provide much needed information for more sharply focused VMMC annual target setting by age bands for these four counties (keeping in mind that effective targeting of specific age groups is often limited more by feasibility of implementation than by data). However, given the data issues experienced before arriving at the estimates, especially for Homa Bay County, a population-based survey is needed to validate male circumcision coverage estimates in the priority counties. This would enable program managers to set better informed targets for attaining and maintaining saturation of VMMC coverage in the priority age groups.

## Supporting information

S1 Table Number of circumcisions double-counted^a^ in each county and year. (DOCX)

S2 Table Percent of voluntary medical male circumcision (VMMC) clients from traditionally non-circumcising communities. (DOCX)

S3 Table Percent of voluntary medical male circumcision (VMMC) clients resident in the county where they were circumcised. (DOCX)

S1 Appendix Number of voluntary medical male circumcision (VMMCs) 2008–2017. Estimates of VMMCs by county and age group from 2008–2017 using unadjusted male circumcision (MC) prevalence estimates (Scenario A), MC prevalence estimates corrected for duplicate reporting alone (Scenario B), MC prevalence estimates corrected for duplicate reporting, population, migration, and replacement (Scenario C), and MC prevalence estimates corrected for duplicate reporting, population, migration, and replacement and with an applied correction factor (Scenario D).
(XLSX)
