# Supplementary material for: Data triangulation to estimate age-specific coverage of voluntary medical male circumcision for HIV prevention in four Kenyan counties
Source: PLoS One. 2018 Dec 18;13(12):e0209385. doi: 10.1371/journal.pone.0209385 (PMC6298728; doi:10.1371/journal.pone.0209385)
Supplement: S1 Table — (DOCX) [file pone.0209385.s001.docx]

**S1 Table. Number of circumcisions double-counted^a^ in each county and year**

| **County** | **2008** | **2009** | **2010** | **Total** |
| --- | --- | --- | --- | --- |
| Homa Bay | 1,452 | 6,925 | 1,303 | 9,680 |
| Kisumu | 1,515 | 8,679 | 587 | 10,781 |
| Siaya | 1,564 | 23,862 | 2,526 | 27,952 |
| **Total** | **4,531** | **39,466** | **4,416** | **48,413** |

**^a^**Double-counting was associated with individual prime implementers and subcontractor that did not operate in Migori.
