# Supplementary material for: Data triangulation to estimate age-specific coverage of voluntary medical male circumcision for HIV prevention in four Kenyan counties
Source: PLoS One. 2018 Dec 18;13(12):e0209385. doi: 10.1371/journal.pone.0209385 (PMC6298728; doi:10.1371/journal.pone.0209385)
Supplement: S2 Table — (DOCX) [file pone.0209385.s002.docx]

**S2 Table. Percent of voluntary medical male circumcision (VMMC) clients from traditionally non-circumcising communities**

| **County** | **2008**  **%** | **2009**  **%** | **2010**  **%** | **2011**  **%** | **2012**  **%** | **2013**  **%** | **2014**  **%** | **2015**  **%** | **2016**  **%** |
| --- | --- | --- | --- | --- | --- | --- | --- | --- | --- |
| Homa Bay | 92 | 97 | 96 | 97 | 96 | 95 | 95 | 96 | 92 |
| Kisumu | 90 | 95 | 94 | 92 | 95 | 91 | 93 | 86 | 90 |
| Migori | 98 | 27 | 96 | 98 | 95 | 92 | 74 | 76 | 98 |
| Siaya | 95 | 98 | 93 | 88 | 99 | 93 | 93 | 92 | 95 |
