# Supplementary material for: Data triangulation to estimate age-specific coverage of voluntary medical male circumcision for HIV prevention in four Kenyan counties
Source: PLoS One. 2018 Dec 18;13(12):e0209385. doi: 10.1371/journal.pone.0209385 (PMC6298728; doi:10.1371/journal.pone.0209385)
Supplement: S3 Table — (DOCX) [file pone.0209385.s003.docx]

**S3 Table. Percent of voluntary medical male circumcision (VMMC) clients resident in the county where they were circumcised**

| **County** | **2008**  **%** | **2009**  **%** | **2010**  **%** | **2011**  **%** | **2012**  **%** | **2013**  **%** | **2014**  **%** | **2015**  **%** | **2016**  **%** |
| --- | --- | --- | --- | --- | --- | --- | --- | --- | --- |
| Homa Bay | 95 | 100 | 97 | 93 | 97 | 94 | 96 | 93 | 98 |
| Kisumu | 96 | 94 | 94 | 89 | 93 | 80 | 95 | 95 | 98 |
| Migori | 100 | 100 | 62 | 72 | 79 | 94 | 96 | 80 | 97 |
| Siaya | 72 | 51 | 55 | 76 | 88 | 96 | 86 | 91 | 82 |
